# Perspectives on deciphering mechanisms underlying plant heat stress response and thermotolerance

**DOI:** 10.3389/fpls.2013.00315

**Published:** 2013-08-23

**Authors:** Kamila L. Bokszczanin, Solanaceae Pollen Thermotolerance Initial Training Network (SPOT-ITN) Consortium, Sotirios Fragkostefanakis

**Affiliations:** ^1^GenXPro GmbH, Frankfurt am MainGermany; ^2^Department of Biosciences, Molecular Cell Biology of Plants, Goethe University, Frankfurt am MainGermany

**Keywords:** thermotolerance, heat stress, pollen, transcriptomic, proteomic, metabolomic, epigenetic

## Abstract

Global warming is a major threat for agriculture and food safety and in many cases the negative effects are already apparent. The current challenge of basic and applied plant science is to decipher the molecular mechanisms of heat stress response (HSR) and thermotolerance in detail and use this information to identify genotypes that will withstand unfavorable environmental conditions. Nowadays X-omics approaches complement the findings of previous targeted studies and highlight the complexity of HSR mechanisms giving information for so far unrecognized genes, proteins and metabolites as potential key players of thermotolerance. Even more, roles of epigenetic mechanisms and the involvement of small RNAs in thermotolerance are currently emerging and thus open new directions of yet unexplored areas of plant HSR. In parallel it is emerging that although the whole plant is vulnerable to heat, specific organs are particularly sensitive to elevated temperatures. This has redirected research from the vegetative to generative tissues. The sexual reproduction phase is considered as the most sensitive to heat and specifically pollen exhibits the highest sensitivity and frequently an elevation of the temperature just a few degrees above the optimum during pollen development can have detrimental effects for crop production. Compared to our knowledge on HSR of vegetative tissues, the information on pollen is still scarce. Nowadays, several techniques for high-throughput X-omics approaches provide major tools to explore the principles of pollen HSR and thermotolerance mechanisms in specific genotypes. The collection of such information will provide an excellent support for improvement of breeding programs to facilitate the development of tolerant cultivars. The review aims at describing the current knowledge of thermotolerance mechanisms and the technical advances which will foster new insights into this process.

## INTRODUCTION

An increasing body of evidence indicates that global climate change is taking place and that it will have important effects on biological processes over the next decades. The expected climate changes include, among other factors, an increase in average temperatures, an increase in atmospheric CO_2_ concentrations and an alteration of wind and rainfall regimes. Based on the “Special Report on A2 and A1B Emissions Scenarios (SRES;” IPCC, 2012), it is predicted that the extreme annual daily maximum temperature (i.e., return value) will likely increase by about 1–3°C by mid-twenty-first century and by about 2–5°C by the late twenty-first century (IPCC, 2012). The current warming trends around the world have already begun to impact agriculture (Lobell et al., 2011). Especially temperature extremes have an impact on a number of different crop species (Hatfield et al., 2011). Thus, for knowledge based breeding and selection strategy for heat tolerant lines an understanding of the molecular thermotolerance mechanisms are more required today than ever before.

In general, plants are simultaneously exposed to various abiotic and biotic stress factors in their natural or agronomic habitats (Ahuja et al., 2010). Plants have evolved to cope with combinations of stress factors responding by complex and often interconnected signaling pathways regulating numerous metabolic networks (Nakashima et al., 2009; Rasmussen et al., 2013). Sets of canonical response genes have been identified to be induced by heat, cold, osmotic or high light stresses (Seki et al., 2002; Rizhsky et al., 2004; Gonzalez-Perez et al., 2011) and in response to pathogen infection and exposure to pathogen-associated molecular patterns (Navarro et al., 2004; Nielsen et al., 2007).

Plants are sessile and thus, most plants have to cope with multiple forms of stress in the same time. Heat and drought represent an excellent example of two general abiotic stress conditions, which in practice often occur simultaneously and in combination have a significantly greater detrimental effect on the growth and productivity of crop plants compared with each condition individually (Craufurd and Peacock, 1993; Savin and Nicolas, 1996; Jiang and Huang, 2001). From this perspective it is conceivable that about 300 cellular stress genes are conserved in all plants analyzed so far to defend or repair vital macromolecules against environmental factors (Kültz, 2005). Physiological characterization of plants subjected to heat stress (HS), drought or a combination of drought and HS reveals that the stress combination has several unique aspects, combining high respiration with low photosynthesis, closed stomata and high leaf temperature (Rizhsky et al., 2002). For example, starch breakdown coupled with energy production in the mitochondria might play a key role in plant metabolism during combined heat and drought stress (Rizhsky et al., 2002, 2004). Further, the level of proline, thought to be important for plant protection during drought stress (Szabados and Savouré, 2010), is strongly suppressed during a combination of drought and HS (Rizhsky et al., 2004). Similar changes in metabolite accumulation were also found, with several unique metabolites, mainly sugars, accumulating specifically during the stress combination (Rizhsky et al., 2004).

In addition to specific reactions, it is well known that overproduction of reactive oxygen species (ROS; **Box 1**) are associated with most forms of stress (Halliwell, 2006) and thus also with HS (Liu and Huang, 2000). As consequence, it is discussed that master regulators of ROS metabolism may provide candidates to monitor stress tolerance in general (Miller et al., 2010). The accumulation of ROS leads to the autocatalytic peroxidation of membrane lipids and pigments, causes alterations in membrane functions and loss of cell semi-permeability (Xu et al., 2011). The hydroxyl radicals can damage chlorophyll, protein, DNA, lipids, and other important macromolecules, which can have detrimental effects on plant metabolism, growth and yield (Sairam and Tyagi, 2004). Thus, protection against oxidative stress injuries is a major challenge for the survival under HS conditions. For that aerobic organisms in general have evolutionary adopted to use ROS as important signal transduction molecule (Mittler et al., 2011). The key to using ROS as signaling molecules appears to be the capacity of cells to detoxify or scavenge them using a network of ROS scavenging enzymes found in almost all cellular compartments (Mittler, 2002; Mittler et al., 2004). However, plant acclimation to a particular stress condition requires a specific response that is tailored to the precise environmental conditions the plant encounters. Thus, a multitude of genes of the ROS gene network were found in the model system *Arabidopsis thaliana* which respond differently to different stress treatments (Mittler et al., 2004), which is in line with a unique acclimation response of plants for each abiotic stress condition. It is further discussed that each combination of two or more different stresses might require a unique response as well (Mittler, 2006). In the following we will focus on the pathways more specific for the HS response (HSR; **Box 1**) and the relation to the reproductive system.

**Box 1. Glossary.**

**Basal (intrinsic) thermotolerance –** an inherent plant ability to survive exposure to temperatures above the optimal for growth, not preceded by acclimation to non-lethal temperature elevations

**Acquired thermotolerance (adaptive) (ATT) –** induced by pre-exposure to elevated but non-lethal temperatures that gives the ability to survive a subsequent severe heat stress that would be lethal in the absence of the preconditioning heat treatment. ATT is transient in nature, and enhances basal thermotolerance and heat endurance via a transition to “efficient” cellular performance when acclimatory homeostasis is reached

**Heat stress response (HSR) –** response to elevated temperatures impairing cell homeostasis by disturbing structural and metabolic integrity of the cell

**Heat Shock Proteins (HSPs) –** proteins accumulated in response to elevated temperatures and function as molecular chaperones in protein folding and protection

**Unfolded Protein Response –** subcomponent of HSR related to protein unfolding in ER and in the cytosol

**Compatible solutes –** low molecular weight molecules, with low inhibitory action on metabolic processes compared to other solutes, acting as osmoprotectants for the maintenance of cell volume homeostasis, but might also have chemical chaperone function

**Reactive oxygen species (ROS) –** Reactive molecules and free radicals derived from molecular oxygen, as by-products of metabolism in mitochondria and other cellular sources with the potential to cause damage to lipids, proteins and DNA when the antioxidant capacity of the cell is exceeded

## THE DEFINITION OF BASAL AND ACQUIRED THERMOTOLERANCE

Plants, like other organisms, exhibit basal thermotolerance (**Box 1**) due to their inherent ability to survive exposure to temperatures above the optimal for growth, but they also have the ability to acquire tolerance to otherwise lethal HS (Larkindale et al., 2005). The ability of plants to respond and successfully acclimate to an episode of severe HS is generally referred to as basal thermotolerance, and is commonly assayed by measuring plant survival following a severe HS episode (Larkindale and Vierling, 2008; Suzuki et al., 2008). Differences between acquired and basal thermotolerance have been documented, e.g., in wheat, where 1314 transcripts are differentially expressed after heat treatments with or without preacclimation (Qin et al., 2008). Certain regulatory and acclimation proteins, such as the transcriptional regulator MBF1c (multiprotein bridging factor 1c; Suzuki et al., 2008) or the ROS detoxifying enzyme catalase, are required for basal thermotolerance but not for acquired thermotolerance (ATT; Suzuki et al., 2008; Vanderauwera et al., 2011). By contrast, some heat shock transcription factors (HSFs), as well as the disaggregating chaperone HSP101, seem to be required for both responses (Queitsch et al., 2000; Liu et al., 2011).

While land plants use constitutively expressed long-term basal heat resistance to withstand the gradual warming of the climate, they also need conditionally expressed short-term acquired thermotolerance (**Box 1**) at the cellular and molecular levels to withstand more acute and frequent heat waves. ATT can be induced by subjecting plants to a moderate level of HS (called “priming”), followed by recovery for a few hours. Alternatively, priming for ATT can also be induced by a slow and continuous rise in temperature as exemplified for *A. thaliana *(Larkindale and Vierling, 2008). Such a gradual rise in temperature most likely mimics natural conditions and appears to be more effective in inducing ATT than the artificial and abrupt treatment (Mittler et al., 2012). The acclimation of *A. thaliana* seedlings to a noxious HS at 45°C was correlated with a massive accumulation of certain transcripts during the priming stages (Mittler et al., 2012), which was found to be much higher during gradual than during abrupt priming treatment. Most of these genes encode for different molecular chaperones such as the small HSPs (sHSPs; HS proteins) and HSP70s, as well as ROS and redox response enzymes, such as ascorbate peroxidase (APX). Thus, while being exposed to initially moderate increases in temperature that gradually develop (Larkindale and Vierling, 2008), plants send an early signal for the timely accumulation of heat shock proteins (HSPs) and metabolites, while also readjusting their pH and redox potentials and reducing their photosynthetic and transpiration activities (Mittler et al., 2012). Together, these mechanisms establish ATT, a transient ability of plants to survive a limited exposure, typically for a few hours, to otherwise lethal temperatures. Remarkably, ATT is usually maintained over a period of approximately 2 days (Charng et al., 2007). Recent advances in genome-wide analyses have revealed complex regulatory networks that control global gene expression, protein modification, and metabolite composition under HS, which will be explored in the next section.

## THE CELLULAR RESPONSE OF PLANTS TO HS

### SIGNAL CASCADES LEADING TO HS INDUCED TRANSCRIPTIONAL REGULATION

Under HS about 5% (~1500 genes) of the plant transcriptome is upregulated twofold or more (Rizhsky et al., 2004; Larkindale and Vierling, 2008; Qin et al., 2008; Finka et al., 2011). A significant fraction of these transcripts encode heat-induced chaperones: 88 out of 1780 in *A. thaliana*, and 117 out of 1509 in wheat (Rizhsky et al., 2004; Qin et al., 2008), which pinpoints to the importance of the HSP-based protection mechanism (Liu et al., 2008; Qin et al., 2008; Ginzberg et al., 2009; Meiri and Breiman, 2009; Perez et al., 2009; Sarkar et al., 2009). The remaining transcripts encode proteins involved in calcium signaling, protein phosphorylation, phytohormone signaling, sugar, and lipid signaling and metabolism, RNA metabolism, translation, primary, and secondary metabolisms, transcription regulation and responses to different biotic and abiotic stresses (Mittler et al., 2012).

The importance of genes despite the ones coding for chaperones for the HSR pathways has been demonstrated in mutants lacking the transcriptional factor *MBF1c*, a highly conserved protein involved in different developmental and metabolic pathways in organisms ranging from yeast to humans (Takemaru et al., 1998; Kabe et al., 1999; Liu et al., 2003). In *A. thaliana* MBF1 is encoded by three genes (*Mbf1a*, * b*, and *c*; Tsuda and Yamazaki, 2004) of which *Mbf1c* (At3g24500) is required for thermotolerance (Suzuki et al., 2008). While MBF1c is not requisite for the regulation of genes encoding HSFA2, different HSPs, or APX 1 during HS, genetic analysis of MBF1c in *Arabidopsis* using gain- and loss-of function mutants demonstrated that MBF1c functions upstream to salicylic acid, ethylene, the pathogenesis-related protein 1 (PR1) and trehalose during HS (Suzuki et al., 2005, 2008). Yeast two-hybrid co-expression analysis provided evidence for the protein–protein interaction between MBF1c and the heat-inducible TPS5 (trehalose phosphate synthase 5; Suzuki et al., 2008) and mutants deficient in TPS5 are thermosensitive (Suzuki et al., 2008). Although MBF1c has been considered as a non-DNA-binding transcriptional co-activator in different systems (Takemaru et al., 1998; Kabe et al., 1999; Liu et al., 2003; Tsuda et al., 2004; de Koning et al., 2009), recently it has been shown that MBF1c binds DNA and controls the expression of 36 different transcripts during HS, including the important transcriptional regulator *DREB2A* (dehydration-responsive element binding protein 2A) that functions upstream to HSFA3 (Schramm et al., 2008), two Hsfs (B2a and B2b), and several zinc finger proteins (including a component of the RNA polymerase basal apparatus, TBP-associated factor 7; Suzuki et al., 2011).

**FIGURE 1:**
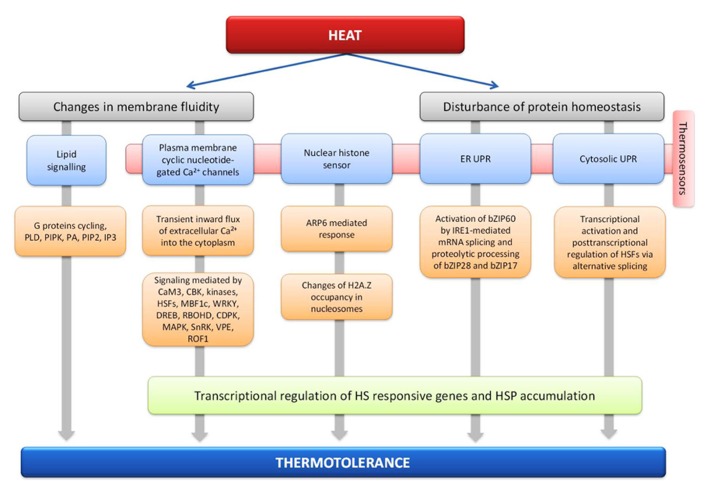
**Plant thermosensors and main signal transduction pathways implicated in heat stress response and thermotolerance**.

The HSR of plants is highly conserved and at least four putative sensors have been proposed to trigger the HSR (Mittler et al., 2012). They include a plasma membrane (PM) channel that initiates an inward calcium flux (Saidi et al., 2009), a histone sensor in the nucleus (Kumar and Wigge, 2010), and two unfolded protein sensors in the endoplasmic reticulum (Che et al., 2010; Deng et al., 2011) and the cytosol (Sugio et al., 2009; **Figure 1**). Each of these putative sensors is thought to activate a similar set of HSR genes leading to enhanced thermotolerance, but the relationship between the different pathways and their hierarchical order remains unclear (Mittler et al., 2011).

#### Plasma membrane channel

It was shown that heat is sensed at the plasma membrane and causes a transient opening of Ca^2^^+^ channels, possibly via modulation of membrane fluidity (Saidi et al., 2009). This would allow a specific inward flux of extracellular Ca^2^^+^ ions from the apoplast into the cytoplasm which is followed within minutes by channel closing despite the ongoing heat-inducing conditions, allowing Ca^2^^+^ pumps to extrude excess Ca^2^^+^ from the cytoplasm (Saidi et al., 2009; Wu et al., 2009). The *A. thaliana* genome encodes over 40 putative calcium channels, many of which are probably located in the PM and might serve as heat sensors (Ward et al., 2009). Many candidate channels display a cytosolic C-terminus with a putative calmodulin-binding domain, raising the possibility that a specific calmodulin (CaM; calcium modulated protein) may be involved in the ensuing steps of the HS signaling pathway. It is proposed that the incoming calcium binds the CaM3 and activates multiple kinases as well as transcriptional regulators of the HSR, such as HSFs, MBF1c, WRKY (Li et al., 2010) and DREB (Zanetti et al., 2003; Suzuki et al., 2011). Increased levels of cytosolic calcium also activate the ROS-producing enzyme RBOHD (respiratory burst oxidases, the ROS-producing enzyme NADPH oxidase), located at the PM, by direct interaction or through activation of a calcium-dependent protein kinases (CDPK) that phosphorylates RBOHD (Suzuki et al., 2011) and can induce multiple mitogen-activated protein kinases (MAPKs; Sangwan et al., 2002). RBOHD-derived ROS can cause membrane depolarization (Miller et al., 2009) or can trigger the ROS/redox signaling network, which would activate downstream pathways via MBF1c, certain HSFs, MAPKs, and/or SnRKs (Mittler et al., 2004; Miller et al., 2009). Recent studies showed increased activity of MPK6 in response to HS and its possible role in the activation of γ vacuolar processing enzyme (VPE), a cysteine protease after HS treatment. It is suggested that HS-induced VPE might exhibit the caspase-1-like activity in heat induced programmed cell death (Li et al., 2012). AtCaM3 can also activate calcium/calmodulin-binding protein kinase (CBK), which regulates the expression of HSPs (Liu et al., 2008). For example, HsfA1a is a suggested *in vivo *target of CBK3 and the regulation of HsfA1a by CBK3 phosphorylation and PP7 dephosphorylation influences HSPs expression and thermotolerance in *A. thaliana* seedlings. Interestingly, ROF1, coding for peptidyl prolyl cis/trans isomerase, is also involved in the calcium dependent phosphorylation of HSFs (Meiri and Breiman, 2009). ROF1 binds to HSP90.1 and is localized in the cytoplasm under normal conditions. Exposure to HS induces nuclear localization of the ROF1-HSP90.1 complex in the presence of the transcription factor HsfA2 that interacts with HSP90.1 but not with ROF1 (Meiri and Breiman, 2009). Moreover, phosphorylation of MBF1c may be a direct or indirect result of CDPK activation as well (Zanetti et al., 2003; Suzuki et al., 2011).

In addition to activating PM ion channels, heat-induced changes in membrane fluidity might trigger lipid signaling. Within minutes of a sudden rise in temperature, plants deploy phospholipids to specific intracellular locations: Phospholipase D (PLD) and a PIPK (phosphatidylinositol 4,5-bisphosphate kinase) are activated, and phosphatidic acid (PA), PIP2 (phosphatidylinositol phosphate kinase), and IP3 (D-myo-inositol-1,4,5-trisphosphate) rapidly accumulate. For this transduction of heat-initiated signal required for PIP2 and PA accumulation, active cycling of a G protein appears necessary. This adaptation response is considered to be similar to that observed during drought stress where PLDα1 mediates ABA stomatal effects through interactions with PP2C and G protein (Mishra et al., 2006). The reduction of phospholipase C9 activity correlates directly with reduced IP3 concentration, down-regulation of sHSPs, and reduced thermotolerance (Zheng et al., 2012). The accumulation of lipid signaling molecules could in turn cause the opening of channels and the triggering of a calcium influx. However, the relationship, if any, between the PM channels that are directly or indirectly activated by heat and the channels that are activated by lipid signaling in plants is unknown. In addition, the order of events in the HS sensing and signaling response is poorly understood.

#### Histone sensor in the nucleus

A screen of *A. thaliana *mutants impaired in heat sensing identified the gene ARP6 as involved in mediating responses to temperature changes (Kumar and Wigge, 2010). ARP6 encodes a subunit of the SWR1 complex, which is necessary for inserting the alternative histone H2A.Z into nucleosomes, while replacing the core histone H2A, and could be involved in temperature sensing (Erkina et al., 2008; Clapier and Cairns, 2009; Erkina et al., 2010). Mutants lacking ARP6 have a reduced content of H2A.Z bound to their chromosomes. Interestingly, the transcriptome of *arp6* null mutants grown at 12°C is similar to that of wild-type plants grown at 12°C and warmed to 27°C, suggesting that H2A.Z-containing nucleosomes can modulate transcription in a temperature-dependent manner (Kumar and Wigge, 2010). Accordingly, in wild-type plants, warming induces a dramatic decrease in H2A.Z occupancy in nucleosomes located at the transcription start site of genes induced by increasing the temperature to 27°C, a process expected to allow nucleosome opening and enhanced transcription of these genes. However, it is unclear whether this mechanism is also required for heat sensing during more classical HS treatments that lead to ATT.

#### ER unfolded protein response

Temperature-induced exposure of hydrophobic residues of proteins may trigger the UPR (unfolded protein response; **Box 1**) in the cytosol and the ER (Mittler et al., 2012). This leads to the accumulation of ER-targeted chaperone transcripts and the activation of brassinosteroid signaling (Che et al., 2010). The UPR may not be as sensitive as the calcium channel PM response because few unfolded proteins are expected to accumulate at low and mild heat stresses. However, in order to cope with the accumulation of unfolded proteins, as consequence of HS, cytoprotective mechanisms have evolved and UPR related to ER is the best characterized in plants (Deng et al., 2013). HSR related chaperones can accumulate in plant cells under non-denaturing conditions in the absence of HS (Saidi et al., 2007), and activation of the UPR seems to require specific calcium signals from the PM (Saidi et al., 2009), suggesting that the UPR is not the primary heat sensor in plants (Mittler et al., 2012). Plants have two UPR signaling pathways, one involving the proteolytic processing of membrane-associated basic leucine zipper domain (bZIP) transcription factors and another involving RNA splicing factor, IRE1, and its mRNA target (Che et al., 2010; Deng et al., 2013). IRE1 is regarded as a dual functional enzyme possessing both serine/threonine protein kinase and endoribonuclease activity (Sidrauski and Walter, 1997). It has been shown that upon HS, IRE1 splices *bZIP60 *mRNA in the cytosol, causing a frameshift leading to the synthesis of a tissue factor without a transmembrane domain, but having acquired a nuclear targeting signal. The spliced form of bZIP60 (bZIP60(s)) is imported into the nucleus to activate UPR target genes (Deng et al., 2011, 2013). Both AtbZIP17 and AtbZIP28 respond to UPR agents and HS (Che et al., 2010) and are activated conventionally in a manner similar to the activation of ATF6 (Liu et al., 2007). Under normal conditions, At-bZIP28 is localized in the ER, but when misfolded proteins accumulate following treatment by ER stress agents, it is translocated to the Golgi where At-S1P (a Ser protease) is localized. At-bZIP28 likely is processed by At-S1P and also by the metalloprotease At-S2P (Che et al., 2010; Liu and Howell, 2010). The released N-terminal component of At-bZIP28 (AtbZIP28n), bearing the transcriptional activation domain and DNA binding domain, relocates to the nucleus where it interacts with CCAAT box proteins composed of nuclear factors (Liu and Howell, 2010). Only recently it has been shown that ATT can be conferred in part by the transcription pathways independent of the HsfA1s in *Arabidopsis*. Although it remains to be seen to what extent bZIP28 is involved in ATT, this membrane tethered transcription factor may act as a sensor of HS in a pathway parallel to that mediated by HsfA1 (Liu and Charng, 2012).

#### Cytosolic unfolded protein response

In contrast to the ER UPR, the cytosolic UPR, which is triggered by the presence of unfolded proteins in the cytosol, in *A. thaliana *is associated with the heat shock promoter element and the involvement of specific HSFs, notably HSFA2, regulated by alternative splicing and non-sense-mediated decay (Sugio et al., 2009). Characterization of *A. thaliana* HSFA2 knockout and overexpression lines showed that HSFA2 is one of the regulatory components of the cytosolic UPR (Sugio et al., 2009). A novel posttranscriptional regulatory mechanism governing HsfA2 expression upon HS has been uncovered. Under severe HS (42–45°C), a new splice variant, HsfA2-III, is generated through the use of a cryptic 5′ splice site in the intron. HsfA2-III can be translated into the small HsfA2 isoform S-HsfA2 during severe HS (42°C for 1 h). S-HsfA2 acts as a functional Hsf and could bind to the TATA box proximal clusters of HS elements (HSE) in the HsfA2 promoter to activate its own gene expression, thus constituting a positive autoregulatory loop (Liu et al., 2013). The S-HsfA2-modulated activation of HsfA2 expression seems not to be mediated by homodimer or heterodimer formation with the known transcriptional activator of HsfA2 (HsfA1d or HsfA1e), although they could bind to the same region of the HsfA2 promoter (Liu et al., 2013).

Apart from *HSFs*, over-expression of other trans-acting factors like *DREB2A* (Yoshida et al., 2011), *bZIP28* (Gao et al., 2008) and *WRKY* proteins (Li et al., 2009, 2010; Wu et al., 2009; Dang et al., 2013) has proven useful in imparting improved thermotolerance (Grover et al., 2013). Plant adaptation to thermotolerance involves also superoxide reductase (SOR), DREB2A, *S*-nitrosoglutathione reductase (GSNOR), and rubisco activase (RCA; Chen et al., 2006; Kurek et al., 2007; Lee et al., 2008; Schramm et al., 2008; Im et al., 2009; Mishkind et al., 2009). For example, RCA is a limiting factor in plant photosynthesis under moderately elevated temperatures and is thus a potential target for genetic manipulation to improve crop plant productivity under HS (Kurek et al., 2007). In turn, the type III alcohol dehydrogenase GSNOR (Achkor et al., 2003) encoded by *HOT5* (Sensitive to hot temperatures 5; Lee et al., 2008) is required for acclimation to high temperature as well as for normal plant growth and fertility. GSNOR acts in plants as well as in other organisms to metabolize *S*-nitroglutathione, which is a mobile reservoir of nitric oxide (NO) in plant cells and links the HSR to NO signaling (Sakamoto and Murata, 2002; Feechan et al., 2005; Rustérucci et al., 2007; Lee et al., 2008). Besides these, up-regulation of expression level of genes coding for proteins involved in osmotic adjustment, ROS removal, saturation of membrane-associated lipids, photosynthetic reactions, production of polyamines, and protein biosynthesis process have yielded positive results in equipping transgenic plants with increased temperature tolerance (Grover et al., 2013). Particular involvement of proteome and metabolome changes occurring as a consequence of the HSR will be explored in next sections of the current review.

### PROTEOME REACTIONS TO HS

Although gene expression analyses have offered insights in understanding the mechanisms underlying HSR and thermotolerance, there is frequently poor correlation between transcript and protein levels in data from heat stressed samples, probably due to alternative splicing and/or posttranslational modifications (Becker et al., 2003; Honys and Twell, 2003). Organ-specific proteomes under HS have been analyzed in various organs of several crop species (reviewed by Kosová et al., 2011). Furthermore, the accumulation or decrease of specific proteins has been documented by comparison of heat sensitive and tolerant vegetative tissues (Kosová et al., 2011), and the significance of certain factors to confer thermotolerance has been confirmed by genetic engineering (reviewed by Grover et al., 2013). In general, these studies confirmed the role and significance of HSP (**Box 1**) in HSR, but also identified other proteins which play an important role for HSR.

Ubiquitins, dehydrins, and late embryogenesis abundant (LEA) proteins are most commonly identified to be induced during HSR and have been proposed to play role in protein degradation, protection against the effects of oxidative damage and dehydration (Ortiz and Cardemil, 2001; Majoul et al., 2003; Wahid and Close, 2007). In addition, several other proteins required for protection against oxidative stress have been found to be accumulated upon HS conditions as well. The most prominent candidates are thioredoxin h (Ferreira et al., 2006; Lee et al., 2007), glutathione *S*-transferase and dehydroascorbate reductase (Lee et al., 2007), cytosolic Cu/Zn-superoxide dismutase (SOD; Herouart et al., 1994) and Mn-peroxidase (POD; Brown et al., 1993). Proteins involved in energy metabolism might also have pivotal functions in HSR and thermotolerance. For example, proteins involved in starch synthesis and degradation were found to be affected by HS. Here, β-amylase which is involved in starch degradation accumulated while glucose-1-phosphate adenyltransferase involved in starch synthesis was down-regulated by HS (Majoul et al., 2004). Thus, the coordinated induction and suppression of specific metabolic pathways, might increase the potential of the cell to survive under unfavorable conditions, by enhancing its energy capacity. In addition, several enzymes involved in tri-carboxylic-acid (TCA) cycle or the pentose phosphate pathway were up-regulated by HS, including UDP-glucose pyrophosphorylase, pyruvate dehydrogenase and transketolase (Lee et al., 2007). In the same study, glycine dehydrogenase, which is implicated in maintaining electron flow and in preventing photoinhibition under stress conditions, was also found to be significantly up-regulated by heat (Lee et al., 2007).

In line with the sensitivity of plastidic function to HS, heat tolerant maize genotypes as well as rice leaves exposed to HS conditions showed increased levels of the plastidic elongation factor, EF-Tu (Bhadula et al., 2001). Similarly, maize mutants lacking the capacity to accumulate EF-Tu at adequate levels are more sensitive to HS (Ristic et al., 2004) and transgenic wheat expressing the maize gene coding for plastidial EF-Tu, exhibited reduced thermal aggregation of leaf proteins, reduced heat injury to photosynthetic membranes and enhanced rate of CO_2_ fixation upon HS, indicating potential chaperone function of EF-Tu (Fu et al., 2008). All results together suggest that EF-Tu plays a role in HS tolerance through the protection of stromal proteins from thermal aggregation (Huang and Xu, 2008) and that the function of plastids is majorly influenced under HS conditions.

#### Protein homeostasis under HS conditions

**FIGURE 2:**
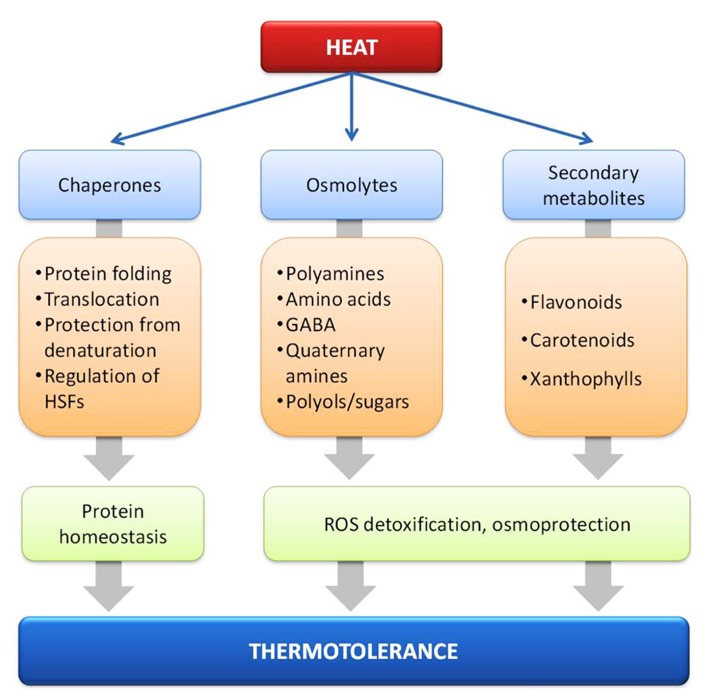
**Molecular chaperones and metabolites such as osmolytes and secondary metabolites implicated in heat stress response and thermotolerance**.

The morphological and functional integrity of cells depends on the equilibrium of most if not all encoded proteins also termed protein homeostasis, which includes the control of synthesis, intracellular sorting, folding, the function and degradation of proteins (**Figure 2**; Hartl et al., 2011). This includes the cytosolic proteins as well as proteins in other cellular compartments. Maintenance of protein homeostasis is prerequisite to ensure optimal growth and development of all organisms under normal and stressful environmental conditions. HS increases the concentration of improperly folded and aggregated proteins, which is assumed to trigger the cellular HSR by activating the transcription of HS induced genes (Morimoto, 1998). The most prominent examples are the classical HSPs coding for molecular chaperones (Boston et al., 1996; Morimoto, 1998).

In all eukaryotic cells the rapid induction of HSR genes and accumulation of HSPs is regulated by the action of HSFs (Scharf et al., 2012). HSFs recognize and bind specific HSE (Nover et al., 2001) in the promoter region of HSR genes with their conserved N-terminal DNA-binding domain (DBD). By sensing the demand of chaperones they are assumed to function as the terminal regulators of proteotoxic stress conditions and their activation is attributed to the formation of oligomeric Hsf complexes, nuclear localization and posttranslational modifications (Morimoto, 1998; Baniwal et al., 2007; Scharf et al., 2012). The importance of several Hsfs for HSR has been confirmed by genetic engineering (Grover et al., 2013). For example, tomato plants overexpressing HsfA1a (Mishra et al., 2002) and *Glycine max* plants overexpressing HsfA1 (Zhu et al., 2006) exhibited increased thermotolerance. Similarly, *Arabidopsis* plants overexpressing *Arabidopsis* and *Lilium longiflorum* HsfA2, tomato HsfA3 and rice HsfA6b exhibit increased thermotolerance as well (Ogawa et al., 2007; Liu et al., 2008; Xin et al., 2010a).

Heat shock proteins are molecular chaperones, which belong to several gene families with distinct functions in the control of protein quality and folding. Under normal growth conditions chaperones of the HSP90, HSP70, and HSP60 families assist folding and sorting of newly synthesized polypeptides in an ATP-dependent manner (Bukau et al., 2006; Hartl and Hayer-Hartl, 2009). Under proteotoxic stress conditions, the cellular chaperone network is complemented by stress-induced members of the same kind as well as of the HSP100 and sHSP families. Together they are involved in protecting misfolded proteins from irreversible aggregation and cooperate in dissolving of protein aggregates or refolding of denatured proteins during stress recovery (Boston et al., 1996; Nakamoto and Vigh, 2007; Pratt et al., 2010; Taipale et al., 2010). The involvement of HSPs has been shown in overexpressing several HSPs in transgenic *Arabidopsis* plants, as well as in other species (Grover et al., 2013). For example, increased thermotolerance has been achieved by overexpressing the plastidial Hsp21 in tomato (Neta-Sharir et al., 2005) and Hsp70-1 in tobacco (Cho and Choi, 2009).

A tremendous importance for the HSR is attributed to the observed HSP-HSF complexes. HSF-chaperone interactions are thought to regulate HSR mechanisms by sensing the demand of chaperones under continuously changing HS conditions as well as possible combinations either with accompanying stress situations, such as drought, salinity, anoxia, UV, and high light, or with subsequently induced secondary stress signals, e.g., of oxidative stress caused by increasing levels of ROS. At the same time, monitoring the state of protein homeostasis by chaperones provides a versatile feedback control system which appears to be directly integrated into the regulation of the cellular HSF activity, not only under stressful but also at normal growth conditions (Hahn et al., 2011).

Cellular homeostasis not only depends on proper folding of proteins but also on proper protein distribution and insertion into different compartments and membranes (Vögtle and Meisinger, 2012) and HS affects the distribution as well (Conde et al., 2011). For example chloroplast precursor proteins accumulate in the cytosol upon HS (Heckathorn et al., 1998) which might be a consequence of the involvement of chaperones in protein transport to the chloroplast surface (Schleiff and Becker, 2011). Under HS conditions the recruitment of chaperones by accumulating denaturated proteins might result in maltargeting or precursor protein aggregation, or both. Thus, the rather unexplored area of maintenance of cellular protein distribution and translocation under HS conditions should be considered for further investigation in the future.

### ALTERATIONS IN METABOLITES DURING HS RESPONSE

In plants, environmental stress generally leads to altered or even disrupted metabolic homeostasis (Wahid et al., 2007). Elevated temperatures can cause a broad range of effects on various plant physiological processes and consequently the levels of several metabolites are altered. Although there is a plethora of information on the metabolite profile of heat stressed plant tissues from targeted metabolomics studies, only few non-targeted studies have been carried out on plants exposed to HS conditions (Kaplan et al., 2004; Rizhsky et al., 2004).

Heat stress causes alterations in hormone biosynthesis and signaling and these changes have been correlated with the HSR and tolerance (Kotak et al., 2007). However, very little is known about the role of plant hormones in HS and thermotolerance, which is remarkable considering the massive research targeted to hormone function in plants. To date it was only reported that exogenous application of the ethylene precursor 1-aminocyclopropane-1-carboxylic acid (ACC), SA, and ABA induced the protection of *Arabidopsis* from heat-induced oxidative damage (Larkindale and Knight, 2002). In the same study, the ethylene-insensitive mutant etr-1, the ABA-insensitive mutant abi-1, and the transgenic line expressing nahG (consequently inhibited in SA production) showed increased susceptibility to heat, suggesting that these hormones have protective role against heat-induced damage. Less attention has been given to the effect of cytokinin in thermotolerance. Cytokinin levels are reduced in response to HS as shown in xylem exudates of heat stressed bean roots (Itai et al., 1973) while application of the synthetic cytokinin, zeatin riboside, reduced HS injury of creeping bentgrass (Liu et al., 2002). Thus, major efforts should be placed to decipher the interplay of the hormone network with the thermotolerance reaction.

Significantly more effort has been placed toward the identification of compounds which might act as antioxidants and osmoprotectants during heat acclimation (**Figure 2**). Heat stressed *Arabidopsis* plants have been found to accumulate several pyruvate- and oxaloacetate-derived amino acids, the oxaloacetate precursors fumarate and malate, some amine-containing metabolites such as β-alanine, γ-aminobutyric acid (GABA), and putrescine, as well as several carbohydrates, including sucrose, raffinose, maltose, galactinol, and myo-inositol (Kaplan et al., 2004). These low-molecular weight organic molecules (amino acids, quaternary amines, and polyol/sugars) that accumulate under stress conditions are collectively called compatible solutes (**Box 1**) and their primary function is the maintenance of cell turgor but they might also act as free-radical scavengers or chemical chaperones, by stabilizing membranes and/or proteins (Hare et al., 1998; McNeil et al., 1999; Diamant et al., 2001).

Proline accumulation occurs in response to different environmental stresses and proline has been demonstrated to have a protective role in some cases. However, proline accumulation in response to HS is plant type specific. While proline levels significantly increase in heat stressed leaves of tomato (Rivero et al., 2004) and tobacco (Cvikrová et al., 2012), the proline content is only slightly altered in heat stressed leaves of chickpea (*Cicer arietinum*; Chakraborty and Tongden, 2005), barley (*Hordeum vulgare*) and radish (*Raphanus sativus*; Chu et al., 1974). Even more, proline did not accumulate in heat stressed *Arabidopsis* plants (Yoshiba et al., 1995; Hua et al., 2001; Rizhsky et al., 2004), and was even reduced in germinating wheat seeds exposed to elevated temperatures (Song et al., 2005). Consistent with the observed differential accumulation of proline is the distinct behavior of transgenic plants with altered expression of proline biosynthetic genes. Overexpression of *Arabidopsis* pyrroline-5-carboxylate reductase gene (AtP5CR) in soybean improved drought and HS tolerance (de Ronde et al., 2000, 2004). In contrast, *Arabidopsis* plants ectopically expressing the Δ(1)-pyrroline-5-carboxylate synthetase 1 gene (AtP5CS1) under the control of the HSP17.6II gene promoter had enhanced proline biosynthesis under HS conditions but a decreased thermotolerance, most likely due to higher ROS production and inhibition of ABA and ethylene biosynthesis (Lv et al., 2011). Thus, the role of proline as protecting agent under HS cannot be globally concluded and might represent one form of species specific adaptation strategies.

The non-proteinogenic amino acid GABA is an osmolyte that has been given special attention. GABA accumulates under different abiotic stresses, including HS (Kinnersley and Turano, 2000). During stress conditions, the cellular levels of Ca^2^^+^ are increased and they are forming complexes with CaM, causing the stimulation of the calmodulin-dependent glutamate decarboxylase activity and GABA synthesis (Kinnersley and Turano, 2000). *Arabidopsis* T-DNA knock-out mutants of the succinic-semialdehyde dehydrogenase (SSADH), the ultimate enzyme of the GABA biosynthesis pathway fail to prevent the accumulation of H_2_O_2_ when plants were exposed to HS, suggesting a protective role for GABA under environmental conditions that cause oxidative stress (Bouché and Fromm, 2004).

Quaternary amines are also involved in plant abiotic stress responses, among them glycine betaine (GB) is the best studied. The levels of GB vary within the plant species and organs under control conditions and many plants such as *Arabidopsis*, tobacco, and rice do not naturally produce GB (Rhodes and Hanson, 1993). Nevertheless, GB production in genetically modified plants increased tolerance to HS (Sakamoto and Murata, 2002). The protective role of GB has been attributed to the prevention of the high temperature-induced association of RCA with thylakoid membranes, allowing the better assimilation of CO_2_ under HS conditions (Yang et al., 2005; Allakhverdiev et al., 2007). GB also increases the activity of ROS scavenging enzymes, thus protecting cellular homeostasis from the accumulation of ROS (Yang et al., 2007).

Sugars accumulate in plants tissues under stress and play a membrane protector role *in planta* (Valluru and Van den Ende, 2008). In sugarcane leaves exposed to HS the increase of pressure potential was correlated with the accumulation of free proline, GB and soluble sugars (Wahid and Close, 2007). Changes in the patterns of genes related to sugar metabolism were observed in *Arabidopsis* double knock-out mutants *hsfA1a/hsfA1b *that show slightly compromised thermotolerance phenotype**(Lohmann et al., 2004; Busch et al., 2005), while increased levels of galactinol and raffinose were found in *Arabidopsis* plants overexpressing HsfA2 (Nishizawa et al., 2008).

Heat stress response and tolerance has been correlated with the accumulation of secondary metabolites such as phenolic compounds including flavonoids and phenylpropanoids (Wahid et al., 2007). During HS, carotenoids like zeaxanthin prevent the peroxidative damage of membrane lipids by ROS (Wahid et al., 2007), while xanthophylls such as violaxanthin interact with membrane lipids and decrease the fluidity of the membrane, conferring thermostability and lower susceptibility to lipid peroxidation (Havaux, 1998). Here, the increase of the activity of phenylalanine ammonia-lyase (PAL), the key enzyme of the phenylpropanoid pathway, is a major mechanism of acclimation of cells to HS (Rivero et al., 2001). Anthocyanins, a class of flavonoids, accumulate during HS in vegetative tissues (Wahid and Ghazanfar, 2006) and reduce the transpirational losses due to the lower leaf osmotic potential (Chalker-Scott, 2002).

## THE CONSEQUENCES OF OUR UNDERSTANDING OF THE HSR FOR THE DAY AFTER TOMORROW

During the past decades many important factors involved in HSR and thermotolerance have been identified on transcript, protein, and metabolite level. Based on these results, some general principles can be extracted (Mittler et al., 2012; Grover et al., 2013) as for example the general response mechanism in form of transcript changes regulated by specific HSFs, the importance of HSPs in maintaining the protein homeostasis and the function of metabolites in signaling and protection. At the same time the detailed analysis has uncovered plant species-specific reactions on all three levels. Thus, the transfer of current knowledge on HSR derived from model plants like *Arabidopsis* can be only executed on the general level, but not for every molecular detail. Compulsory, this calls for the extended analysis of crop plants to define the species-specific variations of the scheme.

Furthermore, recent discoveries included small noncoding RNA species in the regulatory regime of plants’ responses to stress (Sunkar et al., 2007; Ruiz-Ferrer and Voinnet, 2009). The levels of these small RNAs change in response to different abiotic stresses in *A. thaliana*, providing new avenues for the investigation of plant stress signaling (Sunkar and Zhu, 2004). The introduction of high-throughput next generation sequencing technologies has facilitated rapid identification of small RNAs (Fahlgren et al., 2009; Xin et al., 2010b), and several categories are now known to be involved in stress responses, therein heat (Mallory and Vaucheret, 2006; Sunkar et al., 2007; Ruiz-Ferrer and Voinnet, 2009; Xin et al., 2010b; Yao et al., 2010). This information has to be systematically integrated into our current understanding of the HSR.

Moreover, epigenetic mechanisms that are modulated by the environment have been described (reviewed by Mirouze and Paszkowski, 2011) which at the molecular level refer to biochemical modifications of the DNA (e.g., cytosine methylation) and histone proteins (e.g., acetylation, methylation, phosphorylation, ubiquitination) that will mediate the silencing or facilitation of gene transcription by modifying the structure of chromatin. In plants, *de novo* DNA methylation can be modulated in all sequence contexts in regions of RNA–DNA complementarity by the production of small, non-coding, interfering, 21~ 25 nt RNAs (siRNAs) in the process known as RNA-directed DNA methylation (RdDM; Wassenegger et al., 1994; Zhang and Zhu, 2011). In *A. thaliana*, these short RNAs are produced from dsRNA precursors generated by the combined action of the plant-specific DNA-dependent RNA polymerase IV (Pol IV) and RDR2 (RNA-DEPENDENT RNA POLYMERASE 2). DCL3 (DICER-LIKE3) mediates processing of dsRNA to siRNAs, which are loaded into AGO4 (ARGONAUTE 4) effector complexes (Li et al., 2006; Pontes et al., 2006). Only recently it has been shown, that the RdDM pathway is required for basal heat tolerance in *A. thaliana* (Popova et al., 2013). Plants deficient in NRPD2, the common second-largest subunit of RNA polymerases IV and V, and in the Rpd3-type histone deacetylase HDA6 were hypersensitive to heat exposure. Microarray analysis demonstrated that NRPD2 and HDA6 have independent roles in transcriptional reprogramming in response to temperature stress. All genes misregulated in *nrpd2* mutants after HS were located in adjacent to transposon remnants and/or siRNA producing clusters, suggesting that altered heat-responsiveness of protein-coding genes is brought about by a defective epigenetic regulation of nearby transposons in plants deficient in NRPD2. Indeed misexpression of protein-coding genes in *nrpd2* mutants after HS, was related to defective epigenetic regulation of adjacent transposon remnants, which involved loss of control of HS-induced read-through transcription. Thus it has been proven that the transcriptional response to temperature stress, at least partially, relies on the integrity of the RNA-dependent DNA methylation pathway (Popova et al., 2013).

In addition there is growing evidence that ATT and the memory of high temperature exposure may be maintained within an individual and be passed through mitotic (Kvaalen and Johnsen, 2008; Wang et al., 2012) and meiotic divisions (Whittle et al., 2009). The long-term transgenerational memory of ATT is considered to be mediated by stable epigenetic modifications (Angers et al., 2010; Calarco et al., 2012), especially as heat affects chromatin remodeling and nucleosome composition. Furthermore, the role for H2A.Z in temperature sensing has been demonstrated and H2A.Z occupancy is heritable through meiosis (Kumar and Wigge, 2010; Smith et al., 2010). This marks a beginning of the investigation of the epigenetic imprint of HSR, which clearly has to be extended in future.

Finally, taking the experimental observation for manifesting the ATT as guide, where the moderate increase of the temperature has a more pronounced impact on ATT than a short term preheating, it becomes obvious that not a single drastic change but rather moderate global changes are the anticipated alterations to search for. Thus, it will be important to include the reaction of, e.g., organelles or the hormone network into the cascades of HSR to describe the global plant behavior under HS. And last, it will be important to further dissect the HSR in specifically targeted tissues, namely vegetative, and reproductive, as response mechanisms might differ considering the importance for the biological system to be aimed for the reproduction. In this context a special role for “*-omics”* technologies (**Table 1**) is arising as a perspective approach to dissect mechanisms which underpin the systemic functionality on the organismic level.

**Table 1:** “*-omics” *techniques for the investigation of plant HSR.

*“-omics”*	Technique	Reference
Genomics	RAD (restriction site-associated DNA)	Pfender et al. (2011)
	Whole-genome sequencing	Igarashi et al. (2013)
Transcriptomics	RNA-seq	Wang et al. (2009)
	Digital gene expression: MACE (massive analysis of cDNA ends)	Kahl et al. (2012)
	small RNA sequencing	Wei et al. (2011)
	Immunoprecipitation of sRNA-binding proteins	Windbichler et al. (2008)
	RNA– degradome sequencing	German et al. (2008)
Epigenomics	ST-MSDK (methylation-specific digital karyotyping)	Hu et al. (2006)
	Bisulphite sequencing direct	Frommer et al. (1992)
	Nearest neighbor TLC	Ramsahoye et al. (2000)
	MeDIP (methylated DNA immunoprecipitation)	Mohn et al. (2009)
	Anti-mC immunological techniques (5-methylcytosine antibody)	Sano et al. (1980)
	LC– mass spectrometry (LC/MS separation)	Annan etal. (1989)
	HPCE (capillary electrophoretic separation)	Fraga et al. (2002)
	Histone-modification detection (chromatin immunoprecipitation (ChIP) assay)	O’Neill and Turner (2003)
Proteomics	Liquid chromatography/tandem mass spectrometry (LC–MS/MS)	Holmes-Davis et al. (2005)
	Matrix-assisted laser desorption/ionization/time-of-flight mass spectrometry (MALDI-TOF(/TOF)MS)	Zou et al. (2009)
	MALDI-TOF MS, LC–MS/MS	Noir et al. (2005); Dai et al. (2006, 2007)
	Isotope-coded affinity tags (ICAT), LC–MS/MS	Grobei et al. (2009)
	Isobaric tags for relative and absolute quantification (ITRAQ), LC–MS/MS	Petersen et al. (2006)
	Gas chromatography –mass spectrometry (GC –MS)	Lisec et al. (2006)
	Shotgun proteomic approach (Gel-LC-Orbitrap-MS)	Chaturvedi et al. (2013)
Metabolomics	Liquid chromatography (LC)–MS	Halket et al. (2005); Moco et al. (2007), Allwood and Goodacre (2010)
	Capillary electrophoresis (CE)–MS	Monton and Soga (2007)
	Nuclear magnetic resonance (NMR) spectroscopy	Schripsema (2010)

## HS RESPONSE IN POLLEN

Although all plant tissues are susceptible to HS, reproductive tissues are especially sensitive to heat waves, and a few degrees elevation in temperature during flowering time can result in the loss of entire grain crop cycles (Wheeler et al., 2000; Hatfield et al., 2011). Temperature stress can trigger either early or delayed flowering, depending on the species and other environmental conditions. One important modifier is the photoperiod, which provides seasonal information in which a stress can be interpreted in the appropriate context (Craufurd and Wheeler, 2009). Nevertheless, moderate HS will often accelerate flowering, which may cause reproduction to occur before plants accumulate biomass for allocation to developing seeds (Zinn et al., 2010). Crop sensitivity and ability to compensate during later improved weather will depend on the length of time for anthesis. The yield of crops with valuable reproductive structures used for food (i.e., grain crops and horticultural crops) and fiber (i.e., cotton) would be especially sensitive to moderately elevated temperatures projected to result from global climate change. A number of studies utilizing moderately high temperature exposure at different reproductive stages of development have implicated pollen development as the most heat-sensitive process in plant sexual reproduction (Peet et al., 1998; Porch and Jahn, 2001; Sato et al., 2002). For a number of species (e.g., *Phaseolus vulgaris*, *Solanum lycopersicum*, *Vigna unguiculata*, * Capsicum annuum*) the meiotic phase of pollen development has been reported as an exceptionally thermosensitive stage of the reproductive process (Ahmed et al., 1992; Porch and Jahn, 2001; Erickson and Markhart, 2002; Pressman et al., 2002; Sato et al., 2002). In cowpea plants, the stage most sensitive to high temperatures is just after the tetrad stage of pollen mother cell (PMC) meiosis (Ahmed et al., 1992). However, the sensitivities at various stages of growth to high temperature and the resultant effects on the development of PMCs and microspores differ among plant species.

The male gametophyte development starts soon after flower initiation with the formation of reproductive PMCs and differentiation of highly specialized non-reproductive anther tissues (e.g., tapetum (**Box 2**) and stomium). Microsporogenesis (**Box 2**) is initiated by meiosis of PMCs and formation of microspore tetrads, followed by microgametogenesis (**Box 2**) and pollen maturation which includes two subsequent mitotic divisions to form the tri-cellular pollen grain with two spermatocytes embedded in the vegetative pollen cell. Both tapetum and microspore development are essential for male fertility as documented by numerous studies on male sterile mutants (Chen and McCormick, 1996; Aarts et al., 1997; Taylor et al., 1998).

**Box 2. Glossary.**

**Microsporogenesis –** the formation of haploid microspores from diploid mother cells through meiotic division

**Microgametogenesis –** the biological process during which the haploid microspores develop to mature haploid gametes through asymmetric mitotic cell divisions and cell differentiation

**Tapetum –** the innermost cell layer of anther, which gives nutritional support for the developing microspores, but also provides the enzyme catalase required for the release of microspores from tetrads

**Anther dehiscence –** a series of biomechanical processes that lead to the degeneration of stomium and anther opening to facilitate pollen release

**Pollen tube – **a tubular protrusion of the pollen grain, which acts as a carrier of sperm cells to the ovule

**Androgenesis –** process of embryo formation from single, haploid microspores

During reproductive development, plants can respond extremely sensitively to both water deficiency and HS (Barnabás et al., 2008). Pollen grain mitosis 1 and 2 are highly sensitive to elevated temperature both in wheat (Saini et al., 1984) and barley (Sakata et al., 2010). In wheat, two types of abnormal pollen development can be induced by high temperature stress. The first type is caused by tapetal degradation during meiosis. The PMCs complete meiosis but the microspores fail to orient along the periphery of the anther lumen and do not undergo pollen grain mitosis 1 (Saini et al., 1984). Moreover microspores do not have cytoplasm, and may remain immature. In the second case, all the microspores complete the first mitotic division, but only a few of them are able to divide further to develop into normal tri-cellular pollen grains. The rest of the microspores remain immature and do not accumulate starch, so the anthers contain a mixture of fertile and sterile pollen grains (Saini et al., 1984). Without starch to fuel pollen tube (**Box 2**) growth on the stigma, pollen tubes could not reach the ovule (Clément et al., 1994). Moreover, high temperatures can also cause poor anther dehiscence (**Box 2**) characterized by tight closure of the locules, which was shown to reduce pollen dispersal in rice and tomato (*S. lycopersicum*; Matsui and Omasa, 2002; Sato et al., 2002).

It has been revealed that combined drought and heat applied during meiosis of PMCs, in a heat-sensitive genotype of *Triticum aestivum* L. causes serious morphological abnormalities including equal microspore divisions, non-separated tetrads, pollen grains stucking together, microspores developing within the same wall, and multinucleate pollen grains. Pollen grains stuck together presumably by a pollenkitt-like material indicating the malfunction of the tapetum. From the occurrence of multinucleate pollen grains within the anthers of the plants subjected to a combination of osmotic stress and high temperature it was concluded that may be the switch from microgametogenesis to process of embryo formation from single, haploid microspores (androgenesis, **Box 2**) is triggered (Jäger et al., 2008).

### POLLEN-SPECIFIC TRANSCRIPT LEVELS

The transcriptome of different pollen states has been analyzed in some detail. It has been established that the progression from microspores to mature pollen is characterized by large-scale repression of early program genes and the activation of a unique late gene-expression program in maturing pollen (Honys and Twell, 2004). Interestingly, low and high molecular weight HSPs as well as members of the HSF gene family are expressed during microsporogenesis, microgametogenesis and in mature pollen grains in various plant species (Becker et al., 2003; Honys and Twell, 2003; Sheoran et al., 2007; Frank et al., 2009). Members of different classes of cytosolic sHSPs are expressed at higher levels during the early or late stages of pollen development, as well (Bouchard, 1990; Atkinson et al., 1993; Honys and Twell, 2004). In tomato, HsfA2 and Hsp17-CII are highly expressed in the PMCs before the initiation of microspore development and under prolonged HS conditions until mature dry pollen is produced (Giorno et al., 2010). The specific role of these two genes in both development and HSR of tomato pollen cells is still unclear and remains to be elucidated. Furthermore, genes that are related to UPR in ER, such as the ER-resident homologue of Hsp70, BiP and the transcription factor AtbZIP60 are up-regulated during the late stages of pollen as well as tapetum development, suggesting a role for ER stress response in the development and function of pollen and tapetum cells (Iwata et al., 2008).

Analysis of maturing tomato microspores exposed to HS conditions has shown differential gene expression between heat-tolerant and heat-sensitive genotypes prior to applying HS conditions, but not under HS. Higher expression levels of *HsfA2* and *LeHsp17.4-CII* were detected by semi-quantitative RT-PCR analyses in microspores of the heat-tolerant cultivar versus microspores of the heat-sensitive (Frank et al., 2009). Also Pressman et al. (2007) found higher expression levels of *Hsp101* in non-stressed microspores of heat-tolerant compared with microspores of heat-sensitive cultivar. It seems that the capacity for thermotolerance may be achieved by modulating the expression levels of such “responsive” genes prior to HS exposure (Frank et al., 2009). It is supported by the fact that ATT is considered to be achieved by elevating expression levels of “protective” genes prior to HS exposures (Larkindale and Vierling, 2008). In contrast to a heat-sensitive tomato genotype, a heat-tolerant genotype exhibited moderate transcriptional changes under moderate HS (Bita et al., 2011). In the heat-tolerant genotype, the majority of changes in gene expression were represented by up-regulation, while in the heat-sensitive genotype there was a general trend to down-regulate gene expression upon moderate HS. Candidate genes with constitutively higher expression in anthers of the tolerant genotype include the *Hsp82* gene (TC170030), a gene coding for a mitochondrial sHSP, *LeMtHSP* (TC187014), a cathepsin B-like cysteine proteinase (TC171192) and the fructose-1,6-bisphosphate aldolase gene (TC176475). Generally induced upon HS independent of the genotype are several ethylene-responsive genes in developing tomato pollen grains, including *ER5 *(ethylene-responsive late embryogenesis-like protein; U77719), *LeHSC70/ER21 *(ethylene-responsive heat shock protein cognate 70; AF096251), *LeJERF1* (Jasmonate and ethylene responsive factor 1) (AY044235), and *LeMBF1* (ethylene-responsive transcriptional coactivator multiprotein bridging factor ER24; EU240881), as well as a gene encoding ACC synthase (U17972; Frank et al., 2009), pointing to the involvement of ethylene in pollen HSR. Furthermore, 30 h after moderate HS the expression of approximately 1% of the studied transcript-derived cDNA fragments in meiotic anthers of heat-sensitive tomato genotype were altered in their abundance (Bita et al., 2011).

The regulation of gene expression in pollen in response to HS is not yet understood. Only recently in *A. thaliana*, CNGC16, a pollen expressed cyclic nucleotide-gated ion channel (CNGC), has been identified as critical for thermotolerance and pollen fertility under heat as well as under drought stress (Tunc-Ozdemir et al., 2013). CNGCs are Ca^2^^+^-permeable cation transport channels that are activated by cyclic nucleotide monophosphates (cNMPs) and deactivated by binding Ca^2^^+^/CaM (Cukkemane et al., 2011; Ma and Berkowitz, 2011). HS was found to increase concentrations of 3′,5′-cyclic guanyl monophosphate in both pollen and leaves, as detected using an antibody-binding assay (Tunc-Ozdemir et al., 2013). Pollen from *cngc16* mutants exposed to HS showed an attenuated expression of three genes known to be associated with HSR. Two of these marker genes encode for HsfA2 and HsfB1, two Hsfs which are also required for thermotolerance in vegetative tissues (Charng et al., 2007; Ikeda et al., 2011). The third was Bag6, which is one of the target genes under the control of HsfA2 (Nishizawa-Yokoi et al., 2009) and implicated in pollen germination and tube growth in *A. thaliana* (Cartagena et al., 2008). Therefore, this observation provided first insight into the regulation of the transcriptional stress response in pollen, which might involve a link between a stress-triggered cNMPs signal and a downstream transcriptional HSR (Tunc-Ozdemir et al., 2013).

### POLLEN SPECIFIC CHANGES OF PROTEINS UPON HS

Several studies have explored the proteome profile of pollen cells, giving insights into molecular mechanisms involved in pollen development, germination and pollen tube growth (reviewed by Dai and Chen, 2012). For example, it was established that mature pollen grains and seeds from *Arabidopsis* have the same set of major proteins (Grobei et al., 2009). Interestingly, high levels of LEA proteins and chaperones were found, which can be related with the relatively stress-tolerant dormant states for both tissues. The presence of several scavenging proteins related to oxidative stress such as APX, dehydroascorbate reductase, glutathione *S*-transferase, glutathione peroxidase, and monodehydroascorbate reductase in *Arabidopsis* and rice mature and germinating pollen indicate significant function of specific pathways for the maintenance of ROS and redox homeostasis during pollen tube growth (Dai et al., 2007; Zou et al., 2009). The proteome of germinating pollen grains of these two plants are also rich in proteins involved in carbohydrate/energy metabolism and protein synthesis and fate (Holmes-Davis et al., 2005; Dai et al., 2006, 2007; Zou et al., 2009). However, in all these studies late stages of pollen were investigated due to the difficulties of generating enough material for the proteomic analysis of very early stages of pollen development. In contrast, it is assumed that the very early stages are especially prone to stress conditions. In a recent study, cell-specific proteomes from different developmental stages of pollen have been analyzed in tomato including pollen mother cell, tetrad, microspore, polarized microspore, and mature pollen (Chaturvedi et al., 2013). From this study it was revealed that heat shock proteins can be accumulated in the very early stages of pollen development indicating an effect able to protect the cells against sudden abiotic stresses. These observations might hint to a not well understood developmentally controlled key mechanism of protection against environmental stresses called developmental priming (Chaturvedi et al., 2013).

In contrast to the proteomic knowledge, the information about the changes of the pollen proteome under HS is scarce. A comparative proteomic analysis of heat stressed anthers from three rice varieties with different temperature tolerances showed that the tolerant genotype exhibited higher levels of a heat and a cold shock protein compared to the sensitive one and it has been speculated that this accumulation may confer higher heat tolerance (Jagadish et al., 2010).

### POLLEN SPECIFIC ALTERATION OF METABOLITES DURING HS RESPONSE

Changes in the metabolic profile of pollen grain under HS have been recorded in numerous studies, providing more evidence for the mechanisms involved in thermotolerance. In dicots such as tomato and pepper, starch accumulation reaches the maximum at late stages before anthesis and mature pollen grains are starchless, while in monocots the accumulation has a more gradual pattern (Aloni et al., 2001; Pressman et al., 2002). Starch apart from being an energy reserve source during pollen development, may also serve as checkpoint of pollen maturity (Firon et al., 2006). Lower viability of pollen has been also associated with starch deficiency in genetically controlled male-sterile maize mutants (Datta et al., 2002). Under HS, heat sensitive tomatoes accumulate less starch and soluble sugars in pollen than under ambient temperature and this is associated with a decrease of pollen viability (Firon et al., 2006). In tolerant cultivars starch and sugar levels are not affected by HS and hence can be utilized for pollen germination (Pressman et al., 2002; Firon et al., 2006). Decreased levels of soluble sugars in tomato anthers is, at least in part, due to inhibition of acid invertase under HS conditions, which leads to a reduced conversion of sucrose into glucose and fructose (Sato et al., 2006). A similar relation between carbohydrates, HS and pollen viability has been observed in other crops, such as sorghum and barley (Wallwork et al., 1998; Jain et al., 2007). Non-reducing sugars such as sucrose protect the preservation of the native protein structure and the integrity of the cellular membranes during pollen development and especially during the dehydration phase (Firon et al., 2012).

As part of the heat acclimation process, plants produce antioxidants, such as flavonoids, to scavenge ROS produced as a stress response (Wahid et al., 2007). In several species, including tomato, petunia, and maize, flavonoids play an essential role in pollen germination and pollen tube growth (Derksen et al., 1999). In turn, blocking flavonoid biosynthesis through genetic engineering led to male sterility (Schijlen et al., 2007), while flavonoids improve fertilization success under HS conditions as shown in experiments using the *Ipomoea purpurea* chalcone synthase null allele, *α* (Coberly and Rausher, 2003). Consistently, HS conditions led to an increase in total phenolic compounds in tomato pollen, including several classes of flavonoids, suggesting the accumulation of flavonoids as part of the HSR (Georgieva et al., 2000).

Proline which accumulates in response to many different abiotic stresses (Krasensky and Jonak, 2012) is one of the key factors affecting pollen viability (Zhang and Croes, 1983; Lansac et al., 1996). In pollen of several crop plants, such as the legume *Vigna unguiculata* and rice, proline levels decreased upon HS conditions leading to decreased pollen development (Mutters et al., 1989; Tang et al., 2008). Interestingly, the expression of proline transporter 1 mRNA was reduced under HS conditions. This might suggest that proline is incorporated from the tapetum cells into the pollen grains rather than produced within the pollen (Sato et al., 2006).

Polyamines are important for plant defense against environmental stresses in general and their endogenous levels have been proposed to be related with pollen germinability at higher temperatures (Song et al., 1999). The levels of the polyamines spermine and spermidine were reduced in *in vitro* germinating pollen under HS conditions, which led to reduced germination. This reduction of polyamines is due to suppression of the activity of the *S*-adenosylmethionine decarboxylase (SAMDC) during HS (Song et al., 2002). In line, inhibitors of SAMDC led to reduced *in vitro* pollen germination even at ambient temperatures (Song et al., 2002), while addition of spermine and spermidine induced *in vitro* pollen germination and tube growth even at high-temperature (Song et al., 1999).

Remarkably, a reduction of IAA and GA and an increase of ABA was observed in rice anthers upon HS (Tang et al., 2008). This finding is consistent with the observations that plants with impaired auxin biosynthesis exhibit more dramatic injuries at high temperature, while application of auxin completely reversed the male sterility caused by HS in *Arabidopsis* and barley (Sakata et al., 2010). In tomato pollen, a role for ethylene in the HSR and thermo-tolerance has been shown recently (Firon et al., 2012). Here, the ethylene insensitive never ripe (Nr) mutant exhibits an increased heat-stress sensitivity in pollen, while pre-treatment of tomato plants with an ethylene releaser increased pollen quality under HS (Firon et al., 2012). Also brassinosteroids might play a role in pollen thermotolerance in tomato, since addition of brassinosteroids on germination medium under HS increases pollen germination (Singh and Shono, 2005). In addition, the level of cytokinins in maize kernels has been correlated with increased thermotolerance and increased yield stability under HS conditions (Cheikh and Jones, 1994).

Thus, in contrast to the negligible knowledge on the interplay between hormones and HSR for plants in general, significant evidence has been accumulated for the crosstalk in pollen.

## PERSPECTIVES ON DECIPHERING THERMOTOLERANCE MECHANISMS IN POLLEN

The current knowledge of the HSR on reproductive tissues is sparse, but provides foundations essential for the development of breeding strategies based on reliable biomarkers. In addition, the HSR needs to be targeted in crop plants, which can serve as global model, e.g., tomato (*S. lycopersicum *L.). Tomato has long served as a model system for genetic studies because of a series of advantageous characteristics including a diploid, small size genome relatively to other crop species (Arumuganathan et al., 1994). The research on this model is boosted further by the almost fully sequenced and annotated genome (Sato et al., 2012). In addition, tomato is a well established model plant for HS reactions as it displays marked responses to heat, similar to other crop species including pepper, potato, melon, cowpea, wheat, common bean, rice, and barley (Porch and Jahn, 2001; Abiko et al., 2005). Furthermore, tomato is of dietary and commercial importance, but hot summers can result in up to 70% losses in tomato yield (Sato et al., 2004). Thus, especially for field tomatoes heat tolerance is a desired trait. The differences in pollen grain development among the tolerant genotypes are most critical factors to determine fruit set under HS (Levy et al., 1978).

To obtain an understanding of the HSR in tomato pollen or in pollen of any other plant the multi-dimensional signature of HSR including DNA methylation profiles, gene expression data, proteomic compositions and conditions with respect to folding or modification, the cell metabolic status, and the cell integrity has to be described under standard and stress conditions for a multitude of genotypes. At the same time, standard protocols and nomenclatures have to be developed to foster the usability of globally generated data and to compare different lines and species.

To approach these central challenges, the Marie Curie Initial Training Network SPOT-ITN () has been established to investigate fundamental and applied aspects of thermotolerance mechanisms of tomato pollen. The aim of the network is (i) to define common protocols for the analysis of HS effects on pollen, (ii) to establish the HSR at the different levels discussed, (iii) to accompany this information by the analysis of a broad variety of heat-sensitive and heat-tolerant tomato genotypes and mutant lines, and (iv) to analyze these data in the required complexity. The combination of cell biological and molecular strategies with “*-omics”* technologies and bioinformatics can provide valuable information for breeding programs (Langridge and Fleury, 2011). ISOL@ bioinformatics platform conceived to exploit the information generated from the Tomato Genome Sequencing Project for the analysis of the genome organization, the functionality and the evolution of the entire *Solanaceae *family (Chiusano et al., 2008) will serve as platform for data integration. Thus, through transcriptomics, epigenomics, proteomics, metabolomics, and phenomics the footprint of heat tolerance can be identified, especially as HSR is most likely defined by a multidimensional pattern rather than by a single gene.

## Conflict of Interest Statement

The authors declare that the research was conducted in the absence of any commercial or financial relationships that could be construed as a potential conflict of interest.
